# Gender Phenotyping of Patients with Obstructive Sleep Apnea Syndrome Using a Network Science Approach

**DOI:** 10.3390/jcm9124025

**Published:** 2020-12-12

**Authors:** Alexandru Topîrceanu, Lucreția Udrescu, Mihai Udrescu, Stefan Mihaicuta

**Affiliations:** 1 Department of Computer and Information Technology, Politehnica University Timișoara, 300223 Timișoara, Romania; alexandru.topirceanu@cs.upt.ro (A.T.); mihai.udrescu@cs.upt.ro (M.U.); 2 Department I-Drug Analysis, “Victor Babeș” University of Medicine and Pharmacy Timișoara, 300041 Timișoara, Romania; 3 Timisoara Institute of Complex Systems (TICS), 300044 Timisoara, Romania; 4 Department of Pulmonology, “Victor Babeș” University of Medicine and Pharmacy Timișoara, 300041 Timișoara, Romania; stefan.mihaicuta@umft.ro

**Keywords:** obstructive sleep apnea syndrome, gender phenotyping, comorbidities, network medicine

## Abstract

We defined gender-specific phenotypes for men and women diagnosed with obstructive sleep apnea syndrome (OSAS) based on easy-to-measure anthropometric parameters, using a network science approach. We collected data from 2796 consecutive patients since 2005, from 4 sleep laboratories in Western Romania, recording sleep, breathing, and anthropometric measurements. For both genders, we created specific apnea patient networks defined by patient compatibility relationships in terms of age, body mass index (BMI), neck circumference (NC), blood pressure (BP), and Epworth sleepiness score (ESS). We classified the patients with clustering algorithms, then statistically analyzed the groups/clusters. Our study uncovered eight phenotypes for each gender. We found that all males with OSAS have a large NC, followed by daytime sleepiness and high BP or obesity. Furthermore, all unique female phenotypes have high BP, followed by obesity and sleepiness. We uncovered gender-related differences in terms of associated OSAS parameters. In males, we defined the pattern large NC–sleepiness–high BP as an OSAS predictor, while in women, we found the pattern of high BP–obesity–sleepiness. These insights are useful for increasing awareness, improving diagnosis, and treatment response.

## 1. Introduction

Obstructive sleep apnea syndrome (OSAS) is a respiratory disorder during sleep that can range from mild to severe and its prevalence is 5–10% in the general population, regardless of race or ethnicity; many authors consider it an epidemic disease [1,2,3]. OSAS consists of abnormal breathing pauses that occur during sleep, causing sleep fragmentation and excessive daytime somnolence, which produce an impaired life quality, including an increased risk of automobile accidents [4]. OSAS causes the aggravation of cardiovascular diseases [5] (i.e., hypertension [6], arrhythmia [7] and stroke [8], type 2 diabetes [9], cancer [10], and chronic kidney disease [11]. OSAS may increase morbidity and preoperative risks [12]. Because it is associated with many metabolic comorbidities [13,14], OSAS has several distinct clinical phenotypes.

The severity of OSAS is assessed by the Apnea-Hypopnea Index (AHI), measuring the number of apnea and hypopnea events per hour of sleep. (Apnea represents a decrease of at least 90% of airflow from the baseline lasting ≥10 s; Hypopnea represents a drop by ≥30% of pre-event baseline airflow (i.e., nasal pressure) with a duration of ≥10 s and associated with ≥3% oxygen desaturation [15].) An AHI ≥30 characterizes severe OSAS; however, some studies endorse different AHI thresholds for clinically important OSAS, such as 15 (considered as the lower limit for moderate risk), 20 [16], or even 5 (but only when associated with daytime sleepiness or fatigue) [17]. There is a specific variability in scoring respiratory events across different countries [18], especially for mild obstructive sleep apnea, where the clinical relevance and consequences are still uncertain [19].

Although the literature reports gender differences in OSAS evolution [20,21], previous research on OSAS phenotypes—to the best of our knowledge—did not consider gender a factor. We find a recent (2014) successful attempt at phenotyping OSAS comorbidities by Ye et al. [22]; the authors find three clusters: a minimally symptomatic group, a disturbed sleep group, and an excessive daytime sleepiness group. Nonetheless, this study does not indicate significant gender differences between phenotypes.

The study in [17] uses categorical principal component analysis to identify six comprehensive clusters: Healthy but reporting sleep-related symptoms; Mild OSAS without significant comorbidities; Moderate OSAS, obesity, without significant comorbidities; Moderate OSAS with severe comorbidity, obesity, and the exclusive inclusion of stroke; Severe OSAS and obesity without comorbidity and a moderate prevalence of hypertension; and Severe OSAS with severe comorbidities, along with the highest ESS scores and BMI values.

In our previous work [23,24], we employed network science to uncover general (i.e., not gender-specific) phenotypes. In [23], we found the clusters: Severe OSAS group with all considered parameters indicating OSAS risk; Moderate to severe OSAS female group with large NC, high BP, and sleepiness; Moderate OSAS female group with normal NC, obesity, and high BP; Severe OSAS group with age >60 years; Obese young males with large NC, high BP, and with nutritional comorbidities; Moderate OSAS group with large NC; Relatively healthy OSAS male group; Mild to severe OSAS group with high BP and cardiovascular comorbidities.

Many recent studies on OSAS prevalence focus either on general phenotyping/clustering or specific gender differences. However, there is a lack of studies that combine phenotyping with gender analysis. Altogether, the contributions of this paper are:

- We apply independent network-based clustering algorithms on a mixed (i.e., male and female) cohort to uncover gender-specific phenotypes.
- We describe each phenotype and compare them in terms of genders to underline the individual role parameters associated with OSAS.
- We associate phenotypes with comorbidities, highlighting the differences between genders and defining characteristic OSAS development patterns.

Based on the literature, we argue that much research is still required. Any new insight into gender-related differences in OSAS clinical features may increase awareness and improved diagnosis and therapy.

## 2. Experimental Section

### 2.1. Study Design and Participants

The Western Romania (WestRo) cohort consists of 2796 consecutive patients (1948 males and 848 females) with suspicion of sleep breathing disorders, evaluated in four sleep laboratories in Timisoara (Western Romania). We gathered our data between March 2005 and February 2018. We explained the study protocol for each patient at the initial medical visit, described our objective of gathering medical parameter records for further data mining, then obtained the patient’s consent and the referral physicians’ acceptance. Afterward, we performed cardiorespiratory polygraphy and polysomnography (PSG). We carried out polygraphy with both Philips Respironics’ Stardust polygraph (2005) and SleepDoc Porti 7, while PSG was performed with Philips Respironics’ Alice 5 and Alice 6 Diagnostic Sleep System, according to the state-of-the-art guidelines [25]. We performed polygraphy at home and the hospital, and PSG only under medical supervision at the hospital. A senior pulmonologist has manually verified all collected data to ensure information accuracy. Throughout the entire process, we guaranteed complete data confidentiality. Overall, our observational, retrospective study employs only standardized, non-invasive procedures.

We included the 2796 patients with the completed sleep study protocol and signed informed consent in the WestRo cohort, each with 108 anthropometric, medical history, comorbidity, functional, and cardiorespiratory parameters. The data presented in this study are available in the Supplementary Material.xlsx file. The Ethical Committee of Victor Babes Hospital, Timisoara, Romania, approved this paper’s study (approval no. 10/12.10.2013).

### 2.2. Statistical Analysis and OSAS Risk Assessment

We expressed data as a mean ± standard deviation—for continuous variables—and percentages when reporting categorical variables. We coded comorbidities as dichotomous variables by declaring the presence or absence of a condition.

The state-of-the-art scores, such as STOPBang [26], NoSAS [16], and SAS_score_ [24], address the need for OSAS screening. We apply these scores on our cohort to supplement the comparative analysis of each phenotype’s OSAS risks. All the presented scores are computed based on the data available in our WestRo cohort.

The STOP-Bang score combines information about snoring, tiredness, observed apnea, and high BP, with parameters such as BMI, age, NC, and gender. NoSAS provides a good sensitivity for detecting individuals at risk of OSAS [16]. The score (0–17) considers the following parameters: NC (4 points if ≥40 cm), BMI (5 points if ≥30), snoring (2 points if present), age (4 points if ≥55 years), and gender (2 points if male). SAS_score_ [23,24] uses a weighted formula based on BMI, NC, BP, and ESS to quantify the risk of OSAS within the range (1–7).

### 2.3. Network Medicine Modelling

Network science [27,28] brings significant advances in various medical fields such as genomics and drug-target interaction [29,30]. Indeed, network science addresses critical problems in respiratory medicine [31,32].

This paper uses the WestRo cohort data to define two independent patient networks: MPN (male patient network) and FPN (female patient network); in these networks, nodes are patients, either males or females. (We have 1948 males and 848 females.) We create an edge between two nodes if they are compatible according to the parameter classes. We compute the parameter compatibility based on five easy-to-measure parameters with high relevance for OSAS: age group (AG), body mass index (BMI), neck circumference (NC), blood pressure (BP), and Epworth Sleepiness Score (ESS). From these parameters we derive the following parameter classes: age group AG∈{1,2,3,4}, obesity OB∈{0,1}, thick neck TN∈{0,1}, hypertension HT∈{0,1}, and daytime sleepiness SL∈{0,1} (see Equations (1)–(5)).

(1) $$AG=\{\begin{array}{c} 1\;if\;age<20\\ 2\;if\;age\in \left[20,\;40\right)\\ 3\;if\;age\in \left[40,\;60\right)\\ 4\;if\;age\;\geq 60 \end{array}$$

(2) $$OB=\{\begin{array}{c} 1\;if\;BMI\geq 30\\ 0\;otherwise \end{array}$$

(3) $$TN=\{\begin{array}{c} 1\;if\;\left(NC\geq 40\;for\;females\right)or\;\left(NC\geq 43\;for\;males\right)\\ 0\;otherwise \end{array}$$

(4) $$HT=\{\begin{array}{c} 1\;if\;systolic\;BP\geq 140\;and\;diastolic\;BP\geq 90\\ 0\;otherwise \end{array}$$

(5) $$SL=\{\begin{array}{c} 1\;if\;ESS\geq 11\\ 0\;otherwise \end{array}$$

Based on the five parameter classes, the maximum parameter compatibility between two patients is 5. We add an edge between any two patients if they have a parameter compatibility ≥4. Thus, two patients need to have at least 4 out of 5 identical parameter classes (i.e., either healthy or sick) to be considered compatible (and connected by an edge).

We use anthropometric measures to build our network since these are measured objectively and widely accepted in the medical literature [4]. Some alternative studies consider snoring or witnessing apnea episodes as parameters or risk factors, but such parameters are not always observed or measured objectively.

### 2.4. Patient Phenotyping

We apply a dual clustering methodology on the MPN and FPN networks; this dual clusterization consists of modularity-based partitioning [33] (which assigns a cluster identifier to each node), followed by applying of a force-directed layout (i.e., the ForceAtlas2 algorithm [34]). The layout assigns spatial coordinates to each node and, as a result, nodes form topological clusters. We use the Java programming language to process the data and create the networks; the Gephi software package visualizes and processes the networks.

Modularity-based network community detection and force-directed layouts are correlative [35] and complementary, improving the clustering accuracy. We construe the MPN and FPN clusters as patient phenotypes.

## 3. Results

Table 1 presents the clinical parameters, the demographic and anthropometric data of the 2796 patients from the WestRo cohort, divided according to their gender and using the standard deviation (±*SD*) or percentage (%*N*) values. Our male study group consists mainly of middle-aged obese subjects with severe OSAS and daytime sleepiness, whereas our female study group consists mainly of middle-aged obese, hypertensive subjects with moderate to severe OSAS.

**Figure 1:**
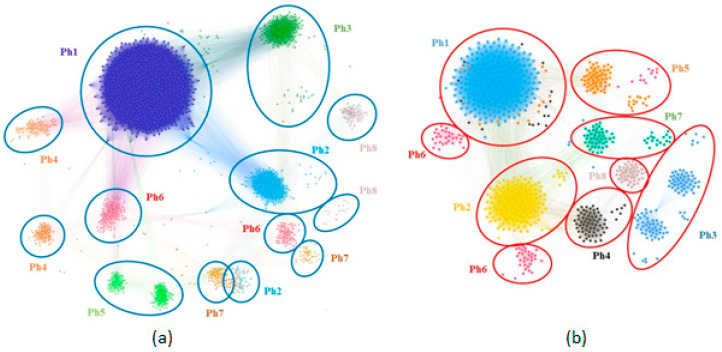
Male and female patient network phenotypes: (**a**) MPN with *N* = 1948 patients and 8 phenotypes; (**b**) FPN with *N* = 848 patients and 8 phenotypes.

The network-based clustering identifies eight phenotypes on the MPN and eight phenotypes on the FPN; they are displayed side by side in Figure 1**.** The figure highlights the clusters in different colors and numbers them from 1 to 8 according to the clusters’ descending size.

**Figure 2:**
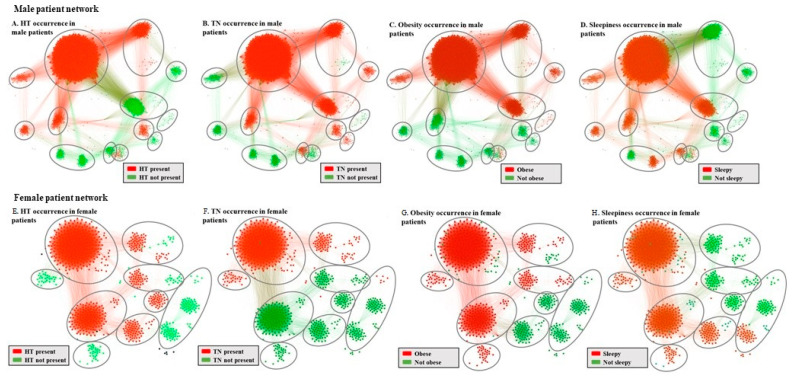
Mapping of obstructive sleep apnea syndrome(OSAS) parameter classes on the MPN and FPN, where ellipses mark each of the eight defined phenotypes, for identifying: (**A**,**E**) Hypertension (HT) presence; (**B**,**F**) Thick neck (TN) presence; (**C**,**G**) Obesity presence; (**D**,**H**) Daytime sleepiness presence in males/females.

We support the visualization in Figure 1. by mapping the parameter classes over the MPN (Figure 2A–D) and the FPN (Figure 2E–H); we can identify phenotypes’ categorization into healthy (green) or sick (red).

We detail male phenotypes in Table 2 and female phenotypes in Table 3. We notice that the highest prevalence is indicated by STOP-Bang (95.63% for males) and the lowest by SAS_score_ (52.51% for males). However, the prevalence of severe OSAS is in between these scores, namely, 70.27%. This observation confirms the purpose of STOP-Bang and SAS_score_; the first one targets preoperative assessment (i.e., calling for higher sensitivity), and the latter aims at population-wide screening (i.e., demanding higher specificity). For the female cohort, the SAS_score_ prevalence (54.59%) is much closer to the severe OSAS prevalence (51.65%); conversely, the STOP-Bang and NoSAS prevalence (87.02% and 68.86%, respectively) are notably higher (i.e., more sensitive), closer to the moderate to severe OSAS (AHI ≥ 15) prevalence (79.60%, see Table 1).

We present the associated comorbidities for males (Table 4. Comorbidities prevalence for phenotypes Ph1–Ph8 in the male cohort (with *N* = 1948)) and females (Table 5) in each phenotype. We assess a total of 10 comorbidities in the WestRo database and find that the most frequent comorbidities for men are coronary artery disease (CAD) (28.3%), nasal septal deviation (NSD) (27.8%), and congestive heart failure (CHF) (24.1%). For women, we find that arrhythmia (AR) (27.8%) and congestive heart failure (CHF) (26.9%) are the predominant comorbidities.

The following parameter class characteristics succinctly describe the eight clusters generated on the male cohort:

- Phenotype Ph1 (severe) corresponds to the sickest subjects that are obese, have a thick neck, high BP, and sleepiness, with coronary artery disease (CAD), congestive heart failure (CHF), nasal septal deviation (NSD), and arrhythmia (AR).
- Phenotype Ph2 (severe without HBP) corresponds to sick male obese patients, have a thick neck, sleepiness, and normal BP, with NSD.
- Phenotype Ph3 (severe without sleepiness) corresponds to sick patients that are obese, have a thick neck, high BP, and no daytime sleepiness, with AR.
- Phenotype Ph4 (moderate hypertensive and sleepy) corresponds to moderately sick patients having a normal NC, high BP, and sleepiness, with NSD.
- Phenotype Ph5 (mild–healthy) corresponds to the healthiest male patients that are not obese, have normal neck, normal BP, and mixed non-significant sleepiness, with CAD and NSD.
- Phenotype Ph6 (moderate not obese) corresponds to moderately sick patients who are not obese and have a thick neck, high BP, and NSD.
- Phenotype Ph7 (mild, thick neck, and sleepy) corresponds to mild OSAS patients that are not obese, have a thick neck, normal BP, and mixed sleepiness, with CAD, NSD, and AR.
- Phenotype Ph8 (mild, thick neck) corresponds to mild OSAS patients that are not obese, have a thick neck, normal BP, and no sleepiness.

We describe the eight clusters on the female cohort by also listing their parameter class characteristics:

- Phenotype Ph1 (severe) corresponds to the sickest obese, thick neck, high BP with sleepiness, significant CAD, CI, and AR female subjects.
- Phenotype Ph2 (severe without HBP) corresponds to sick patients with obesity, normal NC, high BP and sleepiness, and significant CAD.
- Phenotype Ph3 (mild–healthy) has the healthiest female patients with no obesity, normal NC, normal BP and no sleepiness, and without significant comorbidities.
- Phenotype Ph4 (moderate hypertensive) has moderately sick patients with no obesity, normal NC, high BP and mixed sleepiness, and with AR and CAD.
- Phenotype Ph5 (severe without sleepiness) corresponds to sick patients with obesity, thick NC, high BP with no sleepiness, and significant CAD, AR, and CI;
- Phenotype Ph6 (severe with mixed NC) corresponds to sick patients with obesity, mixed NC values, high BP with sleepiness, and without significant comorbidities.
- Phenotype Ph7 (moderate obese and hypertensive) has moderate OSAS patients with obesity, have normal NC values, mixed BP, no sleepiness, and with CAD.
- Phenotype Ph8 (mild hypertensive) has mild OSAS patients with no obesity, normal NC, high BP, no daytime sleepiness, with AR and CAD.

## 4. Discussion

This study represents—to the best of our knowledge—the first attempt to define gender-specific phenotypes and further associate them with comorbidities in OSAS. Our goal was to map the patterns of parameter class association in OSAS by employing a network-driven approach. This study also continues our previous work [23,24] to analyze OSAS using the network medicine approach.

Our study design’s essential feature is that the network-based methodology exploits only easy to measure, objective anthropometric parameters. However, our clustering methodology emphasizes the increased complexity of OSAS phenotypes, from the typically severe (Ph1) to the less obvious parameter class combinations (e.g., male Ph4 with a thick neck and daytime sleepiness). We use modularity clustering when identifying the eight male and female phenotypes [33] combined with a force-directed layout algorithm [34]. Figure 1 shows that the phenotypes obtained with modularity classes are generally consistent with the topological clusters resulting from applying the ForceAtlas2 algorithm. However, upon visual inspection, Figure 1 reveals that some phenotypes are better delimited than others. Specifically, male, moderate-OSAS phenotypes 5, 7, and 8 tend to spatially group and overlap; male mild-OSAS phenotypes 4 and 6 are placed closer together; additionally, female mild-to-moderate-OSAS phenotypes 3, 4, 7, and 8 are partially overlapping. Indeed, the study by Joosten et al. [36] confirms such an overlapping tendency for the mild-and-moderate-OSAS phenotypes. This observation may indicate that the respective phenotypes are firmly related, thus hard to distinguish in clinical practice. Nevertheless, several patients/nodes in these clusters (e.g., in male Ph5 or female Ph3) separate from the overlapping zone; this may indicate we need more patients in the cohort to segregate phenotypes completely. Consequently, we preferred the distinct modularity classes to complement the visual topological clusters because they bring more information and add more detail—which may be useful for medical analysis.

We note that our dual clustering method renders relatively distinct phenotypes for men and women, meaning that different parameter factors associate themselves in specific patterns that characterize the two genders’ clusters. From a medical standpoint, this observation is consistent with several state-of-the-art studies that hold gender as an essential predictor of OSAS severity. For instance, an American study between 2009–2013, conducted on 272,705 patients referred for home sleep apnea testing, concluded that clinical OSAS features are more common in men than women (88.1% older men with OSAS versus 78.7% older women with OSAS, and 68.8% younger men with OSAS versus 42.5% younger women with OSAS) [37]. Additionally, two studies confirm the difference between the genders in terms of AHI distribution and severity—a study performed on 23,806 patients (70% of men with OSAS) [38] and a study with 1010 patients (42.4 average AHI in men versus 33.2 average AHI in women) [39].

**Figure 3:**
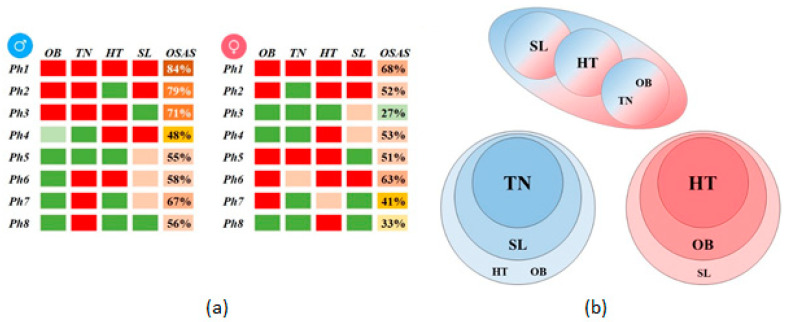
Gender phenotypes comparison: (**a**) Male and female phenotypes described by the four main parameter classes (red–sick, above-high threshold; pink–moderately sick, above-normal threshold; green–healthy, below-normal threshold), and OSAS prevalence (as a percentage); (**b**) Association of parameter classes for the two genders (males–blue, females–red); the upper panel represents overlapping phenotypes, while the lower panel represents gender-specific phenotypes.

For each gender, we obtain eight phenotypes identified by the association of four main parameter classes (obesity OB, thick neck TN, hypertension HT, and daytime sleepiness SL), as depicted in Figure 3a. (For a definition of each categorical parameter class, refer to Equations (1)–(5)). Consequently, we uncover insightful patterns of OSAS development for the two genders through the differential comparison in Figure 3b. We find several identical phenotypes, e.g., the male Ph5 is the same as the female Ph3 (patients with SL). However, we find several phenotypes that do not correspond to the other gender: phenotypes Ph2, Ph6, Ph7, and Ph8 for males and Ph2, Ph7, and Ph8 for females.

We find that four male-specific phenotypes have TN as a typical parameter class; furthermore, 3 out of 4 include SL, one includes HT, and another OB. This result concludes that TN is a significant OSAS predictor for male patients and is associated (in the specified order) with SL, and HT or OB. We find that all three female-specific phenotypes have HT as a standard parameter class. Moreover, 2 out of 3 include OB, and one includes SL, suggesting that HT is a significant OSAS predictor for female patients; HT is associated (in the specified order) with OB and SL.

A limitation of the study is that we clustered patients from a specific geographical area (Western Romania), having mostly Caucasian anthropometric characteristics. Notwithstanding, the dual clustering method itself can be easily employed for other targeted populations to render new parameter class associations, leading to new phenotypes.

Like state-of-the-art [17,22,36], we use only objective, relevant, and easy to measure parameters for the cluster analysis and limit the number of variables that require the subjective observation of a third party—such as witnessed sleep apnea or snoring severity and frequency. Alternately, including additional measurements from the WestRo database would generate more complex (but less robust) phenotypes.

For the WestRo cohort, we measured all the patients under medical supervision with a standardized and validated scale for weight, height, and NC. We measured the systolic and diastolic BP with a standard BP monitor under medical supervision. The diagnostic of systemic high blood pressure considers blood pressure measurements and the patients’ medical history (but may include white-coat hypertension). ESS used alone has known limitations because of its low predictive value for patients with subjective daytime sleepiness; nevertheless, ESS is still the most used sleepiness score in clinical practice worldwide, although usually combined with other objective measurements—as in our study—for better efficiency [40]. In terms of parameter accuracy, we also note that BMI may change over time.

## 5. Conclusions

This paper applies a novel clustering method—based on network medicine—that discovers new gender-specific OSAS phenotypes. This innovative approach—based on assessing five objective patient parameters—identifies eight unique phenotypes for each gender. Some of the detected clusters present common characteristics, regardless of their specific gender (e.g., the severe or mild OSAS phenotypes), while others present a unique pattern of OSAS parameter factor association for both genders. As such, for males, we find that large NC–sleepiness–hypertension/obesity represents a typical association pattern; for women, we find the association pattern hypertension–obesity–sleepiness. We believe that our work will stimulate future research in sleep medicine, help OSAS prediction, and foster a personalized patient management process.

## Figures and Tables

**Table 1:** The WestRo patient cohort, with a total of *N* = 2796 patients, divided into male and female cohorts; we present the synthesis of anthropometric data, clinical parameters, as well as the moderate and severe OSAS prevalence.

Parameter	*Males* (*N* = 1948) Mean/*n* ^1^	*Females* (*N* = 848) Mean/*n* ^1^
Age (years)	51.29 ± 13.22	54.71 ± 12.84
Body-mass index (kg/m^2^)	33.59 ± 6.74	34.09 ± 7.78
Obesity (BMI > 30)	1308 (67.14%)	575 (67.80%)
Neck circumference (cm)	44.83 ± 4.46	39.45 ± 4.36
Thick neck (NC > 43/40)	1458 (74.84%)	367 (43.27%)
Hypertension ^2^	1262 (64.78%)	624 (73.58%)
Systolic BP	176.54 ± 31.28	185.36 ± 32.37
Diastolic BP	105.50 ± 19.50	107.07 ± 20.36
Epworth sleepiness score	11.38 ± 5.40	10.74 ± 5.16
Sleepiness (ESS ≥ 11)	1316 (67.55%)	555 (65.44%)
Mean AHI	46.52 ± 26.36	36.55 ± 25.53
Obstructive apnea	25.96 ± 24.88	19.52 ± 20.43
Central apnea	3.51 ± 5.82	2.02 ± 3.95
Mixed apnea	4.09 ± 7.04	1.97 ± 4.42
Hypopnea	13.35 ± 9.88	12.67 ± 9.63
Moderate OSAS (30 > AHI ≥ 15)	370 (18.99%)	237 (27.95%)
Severe OSAS (AHI ≥ 30)	1369 (70.27%)	438 (51.65%)
STOP-Bang score ≥ 3	1863 (95.63%)	738 (87.02%)
NoSAS score ≥ 5	1757 (90.19%)	584 (68.86%)
SAS_score_ ≥ 3.7	1023 (52.51%)	463 (54.59%)

^1^ The values in Table 1 represent means (with standard deviation *SD*) or counts *n* (with the percentage % from the total *N*). ^2^ We used the maximum hypertension values from the patient’s medical history; see columns “BP max value (sys)” and “BP max value (dia)” in the Supplementary File (Supplementary Material.xlsx).

**Table 2:** Parameter distribution for phenotypes Ph1–Ph8 identified in the male cohort (*N* = 1948).

Parameter	Ph1	Ph2	Ph3	Ph4	Ph5	Ph6	Ph7	Ph8
Size	648 (33%)	249 (13%)	231 (12%)	202 (10%)	194 (10%)	186 (10%)	184 (9%)	34 (2%)
Age	54.8 ± 10.2	45.6 ± 11.2	53.3 ± 11.1	43.2 ± 15.8	56.7 ± 12.1	44.3 ± 13.6	56.8 ± 11.6	37.3 ± 21.8
BMI	37.9 ± 5.9	36.1 ± 5.2	36.5 ± 4.9	29.4 ± 5.2	29.5 ± 5.5	26.9 ± 2.9	28 ± 1.6	28 ± 9.8
NC	47.4 ± 3.6	45.8 ± 3.9	46.4 ± 3.6	42 ± 4.3	39.5 ± 1.8	41 ± 3.8	43.7 ± 1.7	36.6 ± 4.6
SBP	183 ± 28.7	137.3 ± 19.2	185 ± 30.5	131.2 ± 13.5	174.3 ± 28.7	135.3 ± 16.3	176.4 ± 25.4	120 ± 0.0
DBP	109.6 ± 18.4	85.1 ± 19.5	109.1 ± 19	79.5 ± 10.6	104.9 ± 17.5	83.3 ± 11.8	104.5 ± 16	70 ± 0.0
ESS	14 ± 4.7	13.2 ± 4.8	8 ± 4.7	6.5 ± 3.9	11.1 ± 5.5	10.8 ± 4.7	10.3 ± 4.9	7.3 ± 2.2
AHI	56.9 ± 0	56.2 ± 29.8	43.8 ± 23.6	31.4 ± 21.5	36.1 ± 21.3	37 ± 22.4	39.6 ± 19.8	39.6 ± 23.3
Moderate OSAS	74 (11.42%)	37 (14.86%)	44 (19.05%)	53 (26.24%)	55 (28.35%)	49 (26.34%)	46 (25.0%)	8 (23.53%)
Severe OSAS	546 (84.26%)	196 (78.71%)	163 (70.56%)	96 (47.52%)	107 (55.15%)	107 (57.53%)	123 (66.85%)	19 (55.88%)
SB	648 (100%)	246 (99%)	231 (100%)	164 (81%)	192 (99%)	179 (96%)	184 (100%)	4 (12%)
NS	648 (100%)	245 (98%)	227 (98%)	151 (75%)	148 (76%)	132 (71%)	182 (99%)	7 (21%)
SS	593 (92%)	65 (26%)	148 (64%)	1 (1%)	87 (45%)	6 (3%)	119 (65%)	0 (0%)

We present data as the mean ± standard deviation (SD) or percentages (%) of the total *N*. We used the following acronyms: BMI—body mass index, NC—neck circumference, SBP–systolic blood pressure, DBP—diastolic blood pressure, ESS—Epworth sleepiness score, AHI—apnea-hypopnea index, Moderate/Severe-O–patients according to 30 > AHI ≥ 15, and AHI ≥ 30, respectively, SB–OSAS prevalence predicted with the STOP-Bang score, NS–OSAS prevalence predicted with the NoSAS score, SS–OSAS prevalence predicted with the SAS_score_.

**Table 3:** Parameter distribution for phenotypes Ph1–Ph8 identified in the female cohort (*N* = 848).

Parameter	Ph1	Ph2	Ph3	Ph4	Ph5	Ph6	Ph7	Ph8
Size	217 (26%)	150 (18%)	113 (13%)	85 (10%)	76 (9%)	70 (8%)	64 (8%)	58 (7%)
Age	56.6 ± 9.9	56.9 ± 10.3	44.6 ± 18.7	58.6 ± 10.1	55.4 ± 9.5	50.4 ± 10.7	54.3 ± 13.2	61.9 ± 10.4
BMI	40.7 ± 6.3	35.3 ± 4.8	24.6 ± 4.2	27.6 ± 2.3	37.8 ± 6.1	35.6 ± 6.2	35 ± 5.9	26.9 ± 2.5
NC	43.5 ± 3	37.5 ± 1.7	35.1 ± 3.3	37.5 ± 3.2	42.6 ± 2.5	40.6 ± 4.5	36.8 ± 2.4	37.5 ± 3.8
SBP	189.8 ± 30	189.1 ± 29.5	123 ± 14.4	188.3 ± 27.1	192.8 ± 35.2	130 ± 12.5	184 ± 30.7	183.5 ± 25.4
DBP	110.3 ± 18.7	110.2 ± 19.8	72.4 ± 10.4	106.8 ± 17.5	111.7 ± 21.8	76.5 ± 7.8	105.5 ± 22.4	102.9 ± 15
ESS	13.3 ± 4.3	12 ± 5	8.6 ± 5.1	11 ± 4.6	8.3 ± 4.9	12.5 ± 4.7	6.5 ± 3.3	6.6 ± 4.2
AHI	48.2 ± 28.6	36.6 ± 23.3	24.2 ± 25.3	31.1 ± 16.8	35.6 ± 23.8	40.7 ± 25.1	30.8 ± 20.2	27.2 ± 18.9
Moderate OSAS	49 (22.58%)	47 (31.33%)	32 (28.32%)	22 (25.88%)	22 (28.95%)	15 (21.43%)	23 (35.94%)	25 (43.10%)
Severe OSAS	147 (67.74%)	78 (52.0%)	31 (27.43%)	45 (52.94%)	39 (51.32%)	44 (62.86%)	26 (40.63%)	19 (32.76%)
SB	217 (100%)	150 (100%)	44 (39%)	81 (95%)	73 (96%)	63 (90%)	52 (81%)	50 (86%)
NS	215 (99%)	96 (64%)	22 (19%)	51 (60%)	76 (100%)	49 (70%)	32 (50%)	37 (64%)
SS	208 (96%)	116 (77%)	0 (0%)	48 (56%)	47 (62%)	14 (20%)	20 (31%)	9 (16%)

We present data as the mean ± standard deviation (SD) or percentages (%) of the total *N*. We used the following acronyms: BMI—body mass index, NC—neck circumference, SBP—systolic blood pressure, DBP—diastolic blood pressure, ESS—Epworth sleepiness score, AHI—apnea-hypopnea index, Moderate/Severe-O–patients according to 30 > AHI ≥ 15, and AHI ≥ 30, respectively, SB–OSAS prevalence predicted with the STOP-Bang score, NS–OSAS prevalence predicted with the NoSAS score, SS–OSAS prevalence predicted with the SAS_score_.

**Table 4:** Comorbidities prevalence for phenotypes Ph1–Ph8 in the male cohort (with *N* = 1948).

Parameter	Ph1	Ph2	Ph3	Ph4	Ph5	Ph6	Ph7	Ph8
COPD	146 (23%)	39 (16%)	52 (23%)	16 (8%)	29 (15%)	16 (9%)	24 (13%)	0 (0%)
DM	81 (13%)	17 (7%)	24 (10%)	3 (1%)	18 (9%)	2 (1%)	9 (5%)	0 (0%)
CAD	269 (42%)	29 (12%)	85 (37%)	21 (10%)	68 (35%)	10 (5%)	70 (38%)	0 (0%)
CHF	246 (38%)	31 (12%)	76 (33%)	9 (4%)	55 (28%)	11 (6%)	42 (23%)	0 (0%)
AR	180 (28%)	38 (15%)	58 (25%)	17 (8%)	39 (20%)	12 (6%)	49 (27%)	0 (0%)
Stroke	35 (5%)	4 (2%)	18 (8%)	3 (1%)	9 (5%)	0(0%)	8 (4%)	0 (0%)
NSD	192 (30%)	76 (31%)	39 (17%)	59 (29%)	53 (27%)	66 (35%)	56 (30%)	1 (3%)
PP	75 (12%)	32 (13%)	25 (11%)	23 (11%)	18 (9%)	22 (12%)	26 (14%)	1 (3%)
UH	124 (19%)	41 (16%)	42 (18%)	25 (12%)	27 (14%)	27 (15%)	31 (17%)	0 (0%)
AH	90 (14%)	43 (17%)	36 (16%)	31 (15%)	25 (13%)	25 (13%)	22 (12%)	0 (0%)

Acronyms: COPD—chronic obstructive pulmonary disease; DM—diabetes mellitus; CAD—coronary artery disease; CHF—congestive heart failure; AR—arrhythmia; NSD—nasal septal deviation; PP—polyposis; UH—uvula hypertrophy; AH—amygdala hypertrophy.

**Table 5:** Comorbidities prevalence for phenotypes Ph1–Ph8 in the female cohort (with *N* = 848).

	Ph1	Ph2	Ph3	Ph4	Ph5	Ph6	Ph7	Ph8
COPD	24 (11%)	14 (9%)	2 (2%)	5 (6%)	8 (11%)	5 (7%)	2 (3%)	4 (7%)
DM	31 (14%)	9 (6%)	2 (2%)	6 (7%)	10 (13%)	4 (6%)	4 (6%)	3 (5%)
CAD	101 (47%)	73 (49%)	11 (10%)	26 (31%)	31 (41%)	12 (17%)	24 (38%)	19 (33%)
CHF	96 (44%)	57 (38%)	2 (2%)	19 (22%)	23 (30%)	7 (10%)	14 (22%)	10 (17%)
AR	82 (38%)	45 (30%)	10 (9%)	31 (36%)	21 (28%)	11 (16%)	14 (22%)	22 (38%)
Stroke	14 (6%)	8 (5%)	0 (0%)	6 (7%)	7 (9%)	0 (0%)	2 (3%)	2 (3%)
NSD	40 (18%)	22 (15%)	23 (20%)	16 (19%)	5 (7%)	13 (19%)	13 (20%)	14 (24%)
PP	20 (9%)	17 (11%)	17 (15%)	5 (6%)	5 (7%)	11 (16%)	8 (13%)	6 (10%)
UH	28 (13%)	23 (15%)	17 (15%)	16 (19%)	10 (13%)	13 (19%)	8 (13%)	8 (14%)
AH	36 (17%)	22 (15%)	13 (12%)	9 (11%)	15 (20%)	14 (20%)	8 (13%)	10 (17%)

Acronyms: COPD—chronic obstructive pulmonary disease; DM—diabetes mellitus; CAD—coronary artery disease; CHF—congestive heart failure; AR—arrhythmia; NSD—nasal septal deviation; PP—polyposis; UH—uvula hypertrophy; AH—amygdala hypertrophy.

## Supplementary Materials

The following are available online at https://www.mdpi.com/2077-0383/9/12/4025/s1. Supplementary data: Supplementary Material.xlsx.
